# Functional Food in Relation to Gastroesophageal Reflux Disease (GERD)

**DOI:** 10.3390/nu15163583

**Published:** 2023-08-15

**Authors:** Yedi Herdiana

**Affiliations:** Department of Pharmaceutics and Pharmaceutical Technology, Faculty of Pharmacy, Universitas Padjadjaran, Sumedang 45363, Indonesia; y.herdiana@unpad.ac.id

**Keywords:** esophageal disorder, lifestyle modifications, food industry, long-term PPI, processing technology, dietary treatment

## Abstract

Gastroesophageal reflux disease (GERD) is a common esophageal disorder characterized by troublesome symptoms associated with increased esophageal acid exposure. The cornerstones of therapy in this regard include treatment with acid-suppressive agents, lifestyle modifications, and dietary therapy, although the latter has not been well defined. As concerns regarding long-term proton pump inhibitor (PPI) use continue to be explored, patients and healthcare providers are becoming increasingly interested in the role of diet in disease management. However, dietary interventions lack evidence of the synthesis effect of functional foods. The following is a review of dietary therapy for GERD, emphasizing food components’ impact on GERD pathophysiology and management. Although the sequential dietary elimination of food groups is a common practice, the literature supports broader intervention, including reduced overall sugar intake, increased dietary fiber, and changes in overall eating practices. While the primary concern of food companies is to provide safe products, the caloric, nutritional, and functional composition of foods is also generating interest in the food industry due to consumers’ concerns.

## 1. Introduction

Gastroesophageal reflux disease (GERD) is a prevalent condition characterized by troublesome symptoms and esophageal inflammation caused by the reflux of stomach contents [1,2]. Common symptoms include burning chest pain, regurgitation, and difficulty swallowing, while extraesophageal manifestations such as coughing and hoarseness can also occur [3,4,5]. Unhealthy dietary patterns high in fat, sugar, salt, and cholesterol contribute to the increasing incidence of chronic diseases like GERD within the aging global population. In Asia, particularly in Japan, there has been a rapid rise in GERD cases, which are often aggravated by high-fat meals [6]. The complications of GERD include esophagitis, hemorrhage, stricture, Barrett’s esophagus, and adenocarcinoma [7]. GERD is a significant health and social issue that negatively impacts quality of life [8,9,10].

GERD symptoms can be influenced by multiple risk factors [6,11]. Additionally, specific types of food and beverages, such as fast food, tea, oily food, and carbonated drinks, have been associated with an increased prevalence of GERD. Certain conditions like hiatal hernias, pregnancy, lifestyle choices, and certain medications can also trigger acid reflux and increase the risk of developing GERD [12]. Epidemiological studies have revealed several potential risk factors for GERD, including excess adiposity, diabetes, alcohol consumption, and coffee and caffeine consumption [12]. Sociodemographic factors such as age, marital status, and employment status have also been associated with GERD [13].

The treatment options for managing the symptoms of GERD encompass pharmaceutical, surgical, dietary, and lifestyle interventions. The primary approach in treating GERD involves using proton pump inhibitors (PPIs) to reduce acid secretion and reflux. However, previous studies have indicated that a notable proportion of patients (20–30%) continue to experience persistent symptoms despite receiving standard treatment with PPIs. Furthermore, approximately 47.8% of individuals who initially achieved complete resolution of GERD symptoms experienced symptom recurrence after discontinuing PPI therapy. It is worth noting that the long-term use of PPIs has also been associated with an increased risk of foodborne infections. Additional treatment options include acid-suppressive therapy and implementing lifestyle modifications [6].

Exploring non-pharmacological options can lead to better treatment strategies for GERD due to the potential side effects associated with drug therapy [6]. Functional foods refer to food types that provide additional health benefits beyond their basic nutritional function. In the context of GERD patients, functional foods can serve two main purposes, namely, relieving GERD symptoms and improving the digestive process. This treatment method involves modifying one’s diet, which includes reducing one’s consumption of fatty foods and avoiding overeating or eating late at night. Additionally, functional foods are incorporated into this treatment regimen [14]. The emerging technologies in functional foods are paving the way for innovative approaches to enhancing food ingredients’ nutritional value, bioavailability, and functionality. These technologies are revolutionizing the development of functional food products that provide specific health benefits beyond basic nutrition. The findings of this study have the potential to provide valuable evidence for optimizing diet modifications and developing functional foods specifically tailored for patients with GERD [15].

This review aims to analyze the preventive and curative aspects of GERD, with a specific focus on functional foods and their potential in preventing and alleviating GERD symptoms. The review explores various treatment approaches for GERD, considering factors such as anti-inflammatory properties, reductions in acid reflux, the promotion of tissue repair, and the restoration of the esophageal sphincter function. Furthermore, integrating technology into the development of functional foods holds promise with respect to enhancing their effectiveness in treating GERD, thus enabling a personalized approach that caters to individual needs.

## 2. GERD Overview

### 2.1. Pathophysiology of GERD

GERD occurs when the contents of the stomach reflux into the esophagus, causing bothersome symptoms and potential long-term complications. Typical GERD symptoms include heartburn and acid regurgitation, while atypical manifestations encompass noncardiac chest pain, coughing, and laryngitis [16]. Mechanistically, GERD and its associated complications arise when the esophageal mucosa is abnormally exposed to refluxed stomach contents. GERD can arise due to a weakened gastroesophageal barrier, the ineffective clearance of refluxed fluid, or changes in the composition of the refluxed fluid. Repeated exposure to these factors leads to alterations in the integrity of the esophageal lining and the cellular makeup of the tissue [17,18].

**Figure 1:**
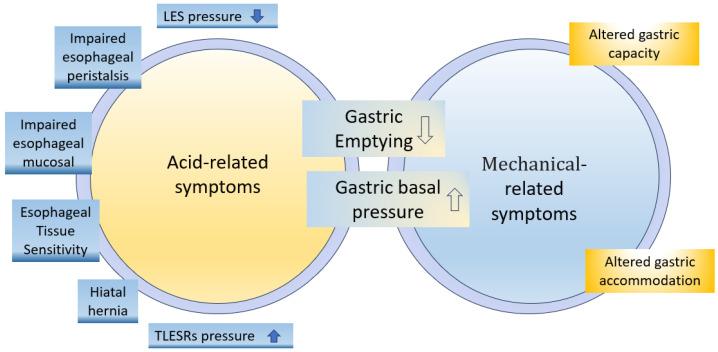
Pathophysiological mechanisms involved in development of upper gastrointestinal (GI) symptoms [19].

The pathogenesis of GERD involves various mechanisms, such as motor dysfunctions, hiatal hernias, and impaired mucosal resistance (Figure 1) [19]. The following factors may contribute to the development of GERD.

1. Lower esophageal sphincter dysfunction

The lower esophageal sphincter (LES) prevents stomach contents from refluxing into the esophagus. It contracts between meals and relaxes during swallowing or transient LES relaxations (TLESRs). People with GERD experience symptoms when the LES relaxes more frequently. LES tone is influenced by neural, hormonal, and dietary factors [6]. TLESRs are triggered by gastric distention from food or air intake, which is influenced by dietary factors and medications. Frequent TLESRs unrelated to swallowing can lead to the reflux of stomach contents into the esophagus due to the higher intragastric pressure than LES pressure in individuals with GERD symptoms [6,18,20,21].

2. Enhanced proximal postprandial gastric acid pocket (PPAGP)

Below the LES lies the PPGAP, where, after a meal, highly acidic stomach contents can accumulate if they do not mix well with the consumed food. In individuals with GERD, the PPGAP is larger, more acidic, and persists longer than in those without GERD. For individuals with a hiatal hernia, the PPGAP can push upwards through the LES, leading to GERD symptoms. Understanding the PPGAP helps explain why some people experience reflux symptoms after eating [22,23,24].

3. Delayed gastric emptying

In approximately 26% of GERD patients, delayed gastric emptying prolongs the retention of acidic food in the stomach, thereby increasing reflux risk. Delayed emptying raises stomach pressure, relaxing the LES and allowing acid into the esophagus, thus contributing to GERD symptoms [17,25]. Peristalsis helps minimize reflux duration by propelling contents upwards, while factors like large food boluses or increased viscosity can slow down contractions. Increased intra-abdominal pressure or food retention inhibits reflux. Refluxed stomach fluid contains irritants like gastric acid, digestive enzymes, and bile salts that can potentially harm the esophageal mucosa. Changes in dietary intake can alter the secretion of these components, and undigested food particles may also contribute to reflux, with varying effects on the underlying mucosa [6,26].

4. Impaired esophageal peristalsis

About 21% of GERD patients are afflicted with impaired esophageal peristalsis, which normally helps clear stomach acid from the esophagus. This reduced clearance of refluxed acid causes the esophageal tissues to be exposed to acidic gastric contents for longer durations, leading to increased damage and more severe GERD symptoms [27,28]. Alongside esophageal peristalsis, the neutralization of gastric acid by salivary bicarbonate plays a protective role in the esophagus. Nevertheless, when peristalsis is impaired, the salivary bicarbonate may not be sufficient to counteract the damaging effects of the refluxed acid [29].

5. Impaired esophageal mucosal defense against the gastric refluxate

The esophageal mucosa is a protective barrier against the substances encountered during GERD. This barrier comprises various structural and functional components that defend against the refluxate, including acidic gastric fluid (hydrochloric acid and pepsin) and alkaline duodenal fluid (bile salts and pancreatic enzymes). Prolonged exposure to the refluxate can breach this defensive barrier, resulting in mucosal damage. In addition to refluxate, drugs can also contribute to esophageal wall damage by directly affecting the esophageal mucosa, creating an acidic or alkaline environment exerting caustic effects. Drug-induced esophagitis can manifest as self-limiting inflammation, but if it persists, it can lead to complications such as severe ulceration, strictures, and, rarely, perforation. It is important to note that GERD can further worsen drug-induced esophagitis, exacerbating the associated symptoms and complications [30,31].

6. Hiatal hernia

A hiatal hernia is often observed in association with GERD and can exist without causing symptoms. However, it plays a crucial role in the development of GERD by interfering with the function of the LES. Research by Patti et al. found that patients with confirmed GERD, regardless of whether they had presented a small hiatal hernia, exhibited similar abnormalities in LES function and acid clearance. However, patients with large hiatal hernias had shorter and weaker LESs, leading to increased reflux episodes. Moreover, the study noted that patients with large hiatal hernias experienced more severe esophagitis. Another study by Ott et al. revealed that 94% of patients with reflux esophagitis had hiatal hernias, highlighting the strong association between the two conditions [32,33].

7. Esophageal Tissue Sensitivity

Individuals with GERD may have heightened sensitivity in their esophageal tissues to even small amounts of acid, resulting in symptoms such as heartburn, chest pain, and regurgitation. Gastric acid increases sensitivity to reflux, enhancing the perception of reflux symptoms. Acid-induced hypersensitivity is more significant in the proximal esophagus due to acid’s damaging effects on the mucosa, impairing barriers and exposing mucosal nerves to toxic refluxate [34].

### 2.2. Complexity of GERD

The complexity of this disease and the multiplicity of its clinical manifestations hinder the development of a singular diagnostic test [35]. Several factors influence the association between diabetes and acid reflux. Firstly, diabetic neuropathy, i.e., nerve damage caused by diabetes, can increase the likelihood of experiencing acid reflux. Additionally, certain medications used for acid reflux treatment, like PPIs, have been linked to a higher risk of developing type 2 diabetes [36,37,38]. Conversely, having type 2 diabetes can also raise one’s likelihood of experiencing acid reflux due to the damage high blood sugar levels inflict on the gastrointestinal tract. Patients with DM are at a greater risk of GERD than those who do not have DM [39]. Obesity, a significant risk factor for both conditions, further strengthens their connection [12].

GERD is common among people with asthma and can worsen and trigger asthma attacks. Treating GERD improves the respiratory symptoms of asthma patients [40]. Possible GERD–asthma symptoms include reflux-related respiratory issues, worsened asthma after meals or lying down, nocturnal asthma, and poor responses to bronchodilators [41]. GERD increases the risk of contracting respiratory infections, including pneumonia, due to refluxate aspiration into the lungs and impaired lung defense mechanisms [42].

Barrett’s esophagus is a condition where the normal lining of the esophagus is replaced by different tissue. It can lead to esophageal adenocarcinoma, a type of cancer, in 3–5% of patients [43]. Barrett’s esophagus develops from progenitor cells at the esophagogastric junction as part of a wound-healing process, replacing damaged squamous epithelium caused by GERD [44].

The oral and maxillofacial manifestations of GERD consist of dental erosion, xerostomia, mucositis, aphthous-like ulcerations, a persistent sour taste, burning mouth, hyperesthesia, bruxism, and temporomandibular disorder [45]. GERD can cause dental problems by exposing tooth enamel to stomach acid, leading to erosion and increased vulnerability to tooth decay [46,47]. GERD can result in tooth sensitivity, enamel erosion, and changes in tooth appearance. Acid reflux can also irritate the gums, causing inflammation, tenderness, and an increased risk of gum disease [48].

GERD disrupts sleep due to nighttime heartburn and regurgitation. Managing GERD through lifestyle changes and medical treatments improves sleep. GERD can cause dysphagia, chronic cough, and sinusitis [2]. Treating GERD and sleep apnea is crucial. Esophagitis associated with GERD requires comprehensive evaluation beyond acid reflux alone [49].

### 2.3. Dietary Factors Pertaining to GERD

The literature on dietary intervention in relation to GERD varies, but some common recommendations can guide patient care [6]. Diet plays a significant role in gastrointestinal health, with certain foods worsening GERD symptoms [6]. High-fat meals, alcohol, chocolate, and carbonated beverages can reduce esophageal sphincter pressure and increase acid exposure [6,50]. Consuming a healthy diet with high fruit and whole grain content, like the Mediterranean diet, may improve GERD symptoms. Improving dietary habits can be a cost-effective strategy with which to reduce the occurrence of GERD instead of relying solely on medication [50].

GERD management entails a comprehensive approach involving optimizing meal sizes, timing, and macronutrient composition. Prioritizing the reduction in meal size, the consumption of simple sugars, and late-night eating helps to mitigate GERD symptoms [6,51]. Caution should be exercised with high-calorie, large-volume, and high-fat meals, as these dietary factors have been linked to exacerbating esophageal reflux [52]. Adopting a slow eating pattern also represents a potential lifestyle modification that may help alleviate GERD symptoms [53]. Scientific evidence supports the notion that fat does not significantly impact esophageal sensitivity to acid [54].

Furthermore, establishing regular eating patterns is crucial to effective GERD management [15,55]. Esophageal acid exposure (EAE) may be more severe after consuming a high-calorie diet than a low-calorie diet with the same fat content. However, patient classification variations and test meal component differences have led to discrepancies between studies [15]. Calorie density plays a role in determining the severity of esophageal acid exposure during GERD after a meal. At the same time, the percentage of fat content in one’s diet significantly impacts the frequency of reflux symptoms [56]. Moreover, a positive relationship exists between high-calorie foods and non-erosive reflux disease (NERD) [57].

Fox et al. found causal links between higher BMI, type 2 diabetes, and an increased risk of GERD. These factors play significant roles in GERD development [12]. GERD is extremely common, and even modest weight gain has been associated with higher symptom burden and objective evidence of reflux observed via endoscopy and physiological measurements [52].

GERD management entails the avoidance of trigger foods like spicy food, citrus fruits, tomatoes, onions, garlic, chocolate, mint, caffeinated beverages, alcohol, and carbonated drinks [50], prioritizing the consumption of lean proteins and emphasizing the intake of whole grains and fiber-rich foods like oatmeal and brown rice instead [6,58]. In one study, individuals with the highest intake of fruits and vegetables demonstrated a 33% lower risk of developing GERD [59]. Additionally, it is recommended that one opts for low-fat dairy products and restricts their consumption of high-fat foods, oils, and fried foods. Adopting a dietary pattern that includes smaller, more frequent meals may help mitigate stomach pressure, while ensuring proper hydration between meals is crucial for effectively managing GERD [60].

The intake of functional foods is very beneficial; for instance, in reference to calcium (dietary calcium intake of 700–1000 mg per day or supplementary calcium intake of 1000 mg per day significantly increases the risk of cardiovascular disease and coronary heart disease [61]), acquiring calcium from dietary sources is generally considered safe and beneficial. Calcium-rich foods include dairy products, leafy green vegetables, fortified plant-based milk alternatives, and certain fish. It is recommended that individuals meet their calcium requirements through a balanced diet rather than relying solely on supplements [62].

In the study conducted by Özenoğlu et al., it was found that diets rich in vegetables, fiber, antioxidants, and caffeine did not exhibit a significant association with an increased risk of dysphagia, which is a symptom commonly associated with GERD [50,63]. GERD is strongly associated with dietary and lifestyle patterns [64]. It positively correlates with alcohol consumption, higher stress levels, education, inadequate sleep, sedentary and physically demanding jobs, nighttime work, lack of exercise, and increased abdominal pressure (e.g., obesity or pregnancy) [57].

### 2.4. Management Treatment of GERD

**Figure 2:**
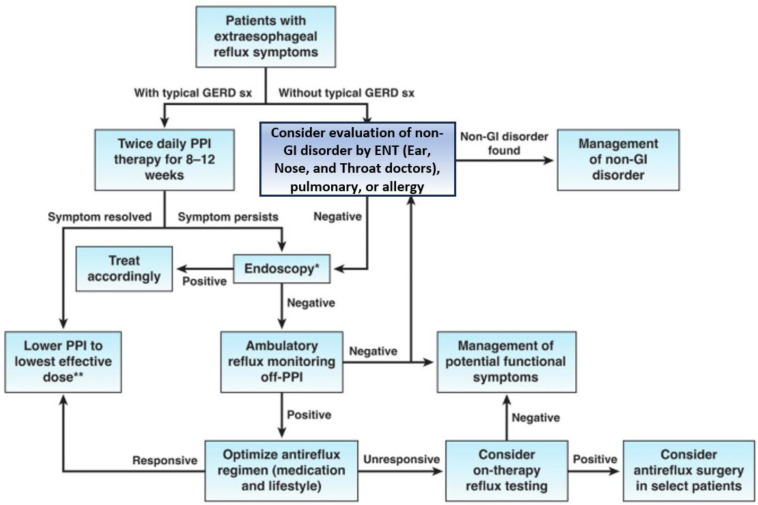
Algorithm for the evaluation of suspected GERD [65]. Reprinted from Clinical Gastroenterology and Hepatology, 21, Chen et al, AGA Clinical Practice Update on the Diagnosis and Management of Extraesophageal Gastroesophageal Reflux Disease: Expert Review, 1414–1421, Copyright (2023), with permission from Elsevier. * Look for evidence of GERD-related injury or complications and rule out alternative esophageal diseases; ** Consider endoscopy and reflux monitoring to support long-term use of PPI.

The Society of American Gastrointestinal and Endoscopic Surgeons (SAGES) has established evidence-based guidelines for managing GERD patients (Figure 2). Nevertheless, additional studies with a low risk for bias are required to further enhance and refine these guidelines [65].

GERD management encompasses various diagnostic approaches, including medical history consideration, physical examination, upper endoscopy, pH monitoring, esophageal manometry, and the barium swallow test. These diagnostic tools help assess the severity of GERD and inform treatment decisions [66].

Lifestyle modification is an important aspect of managing GERD. Avoiding reflux-triggering foods and drinks and eating smaller, more frequent meals can help reduce the chance of reflux. Guidelines suggest that individuals avoid lying down immediately after eating and elevate their heads off their beds by 6–8 inches while sleeping. Maintaining a healthy weight through regular exercise and a balanced diet is also recommended for GERD management [67,68].

Medication plays an important role in managing GERD. The pharmacological targets in this regard have included, firstly, gastric acid neutralization (antacids); secondly, gastric acid secretion reduction (H2 receptor antagonists or PPIs); thirdly, the installation of a physical barrier against refluxate (alginates); fourthly, improved gastric emptying and upper gut motility (prokinetics); and, finally, more recently, in adults, anti-depressant drugs [69,70]. Surgical intervention may be required in severe cases of insufficient medications and lifestyle changes. The most common surgical procedure for GERD is fundoplication, in which the upper part of the stomach is wrapped around the lower esophagus to strengthen the LES and prevent reflux [35]. Surgical treatment is considered a last resort for managing GERD. It is recommended when optimal medical therapy fails, long-term dependence on medication is necessary, the degree of nonadherence to medical therapy is significant, or life-threatening complications occur [71,72,73]. Laparoscopic approaches have become more common and offer similar reflux control and quality-of-life outcomes when compared to open surgeries [74,75]. Robotically assisted laparoscopic surgery for anti-reflux and hiatal hernia procedures is becoming more widespread [76].

Attending regular check-ups with healthcare professionals is important for the effective management of GERD. These appointments allow for the monitoring of symptoms, the evaluation of treatment effectiveness, and the assessment of any complications or progression of the condition.

## 3. Functional Food

### 3.1. Concept of Functional Food

Functional foods are becoming increasingly popular due to the rising cases of chronic diseases and people’s growing interest in healthier eating [5,77]. These foods are attracting attention because they may offer health benefits and meet the demand for better food choices. Functional foods promise to lower the risk of chronic diseases and enhance overall physical and mental well-being [5]. While the exact definition of a functional food is under debate, this concept drives research and innovation in developing safer and nutrient-rich options that contribute to ensuring better health and disease prevention. The European Food Safety Authority defines functional foods as foods that transcend basic nutrition and positively impact certain bodily functions, promote health and well-being, and/or reduce the risk of disease [78].

Functional foods can help improve quality of life and reduce the risk of serious illness or delay its onset [79]. Functional foods are natural foods with added health-promoting ingredients or that have been modified to enhance their benefits [80]. They can be natural or processed, and their positive effects should be considered in terms of the amounts typically eaten as food [78].

Functional foods can be categorized based on composition and purpose (Kamerow 2004). The types of functional foods organized in reference to the above qualities include the following:

1. Conventional foods containing natural bioactive substances that naturally contain beneficial compounds, such as oat beta-glucan, fruits, and vegetables rich in lycopene and lutein.
2. Foods modified via enrichment with bioactive substances: These foods are regular foods fortified or enriched with specific bioactive components, such as margarine with added phytosterols, calcium-fortified orange juice, or folic-acid-rich pomegranates.
3. Foods intended for medicinal use, which are specialized formulae that resemble foods but are designed to be consumed as medicine via and according to a prescription, such as special formulas for children with medical conditions.
4. Special dietary needs foods, which are formulated for specific requirements (gluten-free, lactose-free, and infant food).

Companies must comply with specific requirements and understand complex regulations and consumers’ demands [81]. Before endorsing the marketing of the suggested functional foods, more high-quality evidence is required in several key areas, which are indicated below:

1. The identification of appropriate active ingredients: This entails a comprehensive investigation into the suitable active ingredients or combinations thereof, particularly when dealing with groups of related substances or the diverse bacteria used in probiotics.
2. The determination of optimal concentrations: Rigorous research is essential to ascertain the optimal concentrations for the active ingredients present in functional foods.
3. A concrete demonstration of health benefits: The claims should be attributed to functional foods. A rigorous and thorough demonstration of the purported health benefits must be executed through well-designed research.
4. Thorough assessment of safety: Before marketing, a meticulous assessment of the safety of functional foods is indispensable in order to ensure consumer well-being and confidence [14].

### 3.2. Importance and Popularity of Functional Food

Poor lifestyles, including those characterized by unhealthy nutritional habits and a lack of physical activity, lead to an increase in the development of degenerative diseases and even death [82]. It has been estimated that 10–30% of the Western population suffers from degenerative diseases, including GERD [83]. This situation has forced people to prioritize their health, diet, and well-being. As a result, awareness about the relationship between food and health is increasing [84]. The demand for functional foods is also increasing, as these foods offer more health benefits, such as reducing fatigue and boosting immunity, and enhanced nutrition. Functional foods are important with respect to promoting health and preventing disease [85]. This growth is driving the rapid growth of the global functional food market, which is expected to amount to USD 276.68 billion by the end of 2024, with a CAGR of 9% [86].

With regard to the development of functional foods, there are five determinants of consumer acceptance of functional foods: product characteristics, sociodemographics, and psychological, behavioral, and physical factors [87,88]. Various factors, such as lifestyle, age, sex, personality, income, education, ethnicity, traditions, beliefs, physiological factors, sensory preferences, marketing, and available information, influence food choices [5,89]. Understanding these factors is important for developing new functional foods in a competitive market. By considering these categories, experts and marketers can make informed decisions in order to increase the acceptance and adoption of these products.

The FDA regulates all claims associated with food products, including those regarding product labels, websites, or advertisements. Functional food labels can make four types of FDA-regulated claims: nutrient content claims, authorized health claims, qualified health claims, and structure–function claims. Structure–function claims do not require post-market FDA review, while authorized and qualified health claims have specific requirements and scientific support. The FDA ensures that these claims provide accurate and comprehensive information to consumers [90].

### 3.3. Key Characteristics and Benefits of Functional Food

Functional foods are distinguished by their high nutrient density, offering a concentrated amount of essential nutrients per serving. They are packed with vitamins, minerals, fiber, antioxidants, and other bioactive compounds, allowing individuals to obtain their nutritional requirements while consuming smaller quantities. People can optimize their nutrient intake and support their overall health by including functional foods in their diets [91,92]. Functional foods can be categorized into various types based on their desired characteristics and benefits:

1. Fortified foods: Fortified foods are supplemented with additional nutrients to enhance their nutritional value, thereby addressing nutrient deficiencies and improving overall nutrition [93].
2. Enriched foods: Enriched foods are designed to replenish nutrients lost while processing certain foods. An example is refined grains, like white flour, where vitamins and minerals are added to the product to compensate for the nutrients lost during the refining process [94].
3. Modified foods: Modified foods undergo specific alterations to provide health benefits beyond basic nutrition. They include products with reduced fat or sugar content, gluten-free products, or low-sodium options [95].
4. Naturally functional foods: Naturally functional foods are inherently rich in beneficial compounds and offer health benefits without requiring any modifications. Examples include fruits, vegetables, whole grains, nuts, and seeds. These foods naturally contain vitamins, minerals, antioxidants, fiber, and other bioactive compounds that promote health and well-being.

### 3.4. Preparation in Functional Food

The search for functional food involves identifying bioactive compounds from various sources, such as plants, fungi, or animals. Through careful identification, assessing bioavailability, determining safe doses, and finding relevant biomarkers, we can understand these compounds’ roles in promoting health. This knowledge will formally define functional foods, reinforcing their significance in improving well-being [96]. Thorough research, testing, and regulation are required to ensure the efficacy and safety of functional food products. The growth and innovation of various functional food preparations provide consumers with more effective and personalized choices that support their health and well-being.

Emerging technologies offer various approaches to enhancing functional foods, including the following:

1. Removing harmful components that can induce adverse effects when consumed (e.g., allergenic proteins) [97,98].
2. Increasing the concentration of beneficial components in foods [99].
3. Replacing excessive or unhealthy components (usually macronutrients) with components with positive effects [100,101,102].
4. Improving the bioavailability or stability of recognized functional components or reducing the potential risk of disease [102,103,104].
5. Improving food processing and drying foods such as fruits can reduce the amount of vitamin C they retain, but it can also concentrate other nutrients [105,106,107]

## 4. Mechanism of Functional Food in GERD Mitigation

Functional foods can play a beneficial role in managing GERD by providing relief from symptoms and promoting overall digestive health. While they should not replace conventional medical treatments, incorporating certain functional foods into one’s diet may help alleviate GERD symptoms.

### 4.1. Reducing Acid Production

Functional foods can be valuable additions to the diets of individuals with GERD. These foods can help manage GERD symptoms and reduce the frequency and severity of acid reflux episodes. Firstly, incorporating fiber-rich foods such as whole grains, fruits, vegetables, and legumes can aid in promoting healthy digestion and preventing constipation, the obverse of which can contribute to GERD symptoms [108].

Popular writings have suggested that certain foods impact stomach acidity and relieve heartburn symptoms, although the scientific research supporting these claims is often limited. However, some evidence suggests the potential benefits of specific foods for managing acid reflux and heartburn. Aloe vera has traditionally been used due to its soothing effects on the digestive system, and consuming small amounts of pure aloe vera juice before meals may reduce acid reflux symptoms. Aloe vera contains enzymes that can help break down sugars and fats and help maintain the smooth functioning of the digestive system [109]. Yogurt, when not too sour, is beneficial for acid reflux due to its probiotics, which aid in normalizing bowel function. Additionally, yogurt provides protein and helps soothe stomach discomfort, often providing a cooling sensation [110]. Low-fat yogurt is particularly effective in maintaining intestinal barrier integrity compared to nonfat yogurt when exposed to pro-inflammatory cytokines [111]. Additionally, low-fat or lean protein sources like skinless poultry, fish, and tofu can help minimize symptoms, as high-fat foods can relax the LES and trigger acid reflux. Oats are high in fiber, which helps regulate digestion and reduce the risk of acid reflux [112]. Oatmeal is often well-tolerated and can exert a soothing effect on the stomach lining.

Bananas are considered low-acidity foods and may help neutralize stomach acid [113]. Bananas are alkaline and rich in pectin, a soluble fiber that helps keep food flowing adequately through the digestive tract, thus preventing food stasis in the stomach for prolonged periods, which helps limit acid production and reduces the likelihood of acid reflux. A fiber-enriched diet helps to control symptoms [114] and improves esophageal motility in patients with NERD [108]. Fresh bananas effectively generate a protective coating on the esophageal mucous lining, strengthening the mucosal defenses against reflux. Almonds, a good source of healthy fats, can neutralize stomach acid and be consumed as a snack or added to meals. Green leafy vegetables such as spinach, broccoli, kale, asparagus, and Brussels sprouts are naturally low in fat and sugar. Ginger has been traditionally used because of its anti-inflammatory properties, which may help inhibit gastric acid production [115,116]. The prokinetic activity of ginger has been confirmed in in vitro and in vivo tests [117]. Additionally, licorice root contains compounds that can inhibit an enzyme called H+/K+ ATPase, which is involved in acid secretion.

These foods may help reduce stomach acid secretions due to their alkaline nature. A combination of various natural active ingredients (hyaluronic acid, Altea, Malva, apple, aloe vera, L-tryptophan, calcium gluconate, sodium bicarbonate, and Musa paradisiaca) have shown promise as alternative or complementary treatments for patients with NERD. These formulations have demonstrated the ability to significantly reduce the frequency and intensity of symptoms, as assessed using the reflux disease questionnaire (RDQ) [118].

### 4.2. Soothing and Protecting the Esophagus

Some functional foods possess soothing properties that can help alleviate irritation and protect the delicate lining of the esophagus from stomach acid. For example, marshmallow root and slippery elm contain mucilage, which can form a gel-like coating on the esophageal lining. This coating acts as a barrier against acid, reducing discomfort and inflammation. In one study, a fiber-rich diet led to notable improvements in NERD patients, including increased minimal LES (lower esophageal sphincter) resting pressure, reduced gastroesophageal reflux, and decreased weekly heartburn frequency [108].

### 4.3. Enhancing Digestive Function

Functional foods that support healthy digestion can indirectly alleviate GERD symptoms. Probiotics, such as those found in yoghurt and fermented foods, promote a healthy balance of gut bacteria, thereby promoting a healthy gut environment and aiding digestion. [119,120]. Probiotics have been found to offer benefits in cases of small-scale intestinal bacterial overgrowth by affecting immunity and intestinal motility under different conditions [120]. Probiotic-rich foods like yoghurt, kefir, sauerkraut, or kimchi may help maintain a balanced gut microbiome, improve digestion [121,122], and benefit individuals with GERD. This can help improve overall digestive function and reduce the occurrence of acid reflux [123].

Fiber-rich foods, like whole grains, fruits, and vegetables, aid in proper bowel movements and prevent constipation [124], which can contribute to GERD symptoms [58,125]

Papaya contains enzymes such as papain that aid digestion and help break down proteins [126,127]. Eating ripe papaya or drinking papaya juice can support healthy digestion [127] and reduce symptoms of GERD [116].

### 4.4. Alleviating Inflammation

Chronic inflammation in the gastrointestinal tract can worsen GERD symptoms [116]. Some functional foods possess anti-inflammatory properties that can help reduce inflammation and provide relief. Turmeric contains curcumin, a compound with potent anti-inflammatory effects [128,129]. Consuming turmeric or curcumin supplements may help reduce inflammation in the esophagus and stomach lining [130]. Foods rich in omega-3 fatty acids, such as fatty fish (e.g., salmon) or flaxseeds, can also help reduce inflammation [131]. Omega 3 anti-inflammatory effects are triggered through the activation of their receptor, free fatty acid receptor 4 (FFAR4) [132]. In particular, FFAR3 and FFAR4 may have diagnostic and therapeutic 500 potential in relation to GERD [133].

Chamomile tea has calming properties and can help reduce inflammation [133]. It may relieve GERD symptoms when consumed after meals or before bedtime. Chamomile (*Matricaria chamomilla* L.) has been a highly popular family herb since antiquity; it is generally used to relieve nervous excitability and digestive disorders, stomach cramping, dyspepsia, and flatulence [117]. Ginger has natural anti-inflammatory properties [134] and can help soothe the gastrointestinal tract [121]. It may reduce the frequency and severity of heartburn episodes [116]. Ginger can be consumed as ginger tea, added to smoothies, or used in cooking. The anti-inflammatory and analgesic effects of ginger essential oil validate the traditional uses of ginger root in treating inflammatory diseases of gastrointestinal tracts [122,134,135]. One study found that Black Garlic (BG) supplementation increased catalase levels and tended to increase superoxide dismutase levels in the esophagus. The effects of BG were better than those of raw garlic, suggesting that BG treatment may alleviate esophagitis by regulating NF-κB-mediated inflammation [136].

## 5. Perspective

Technological advances are important with regard to advancing functional foods, as they synergistically facilitate their development. Moreover, exploring functional compounds’ dosage, safety, stability, delivery systems, and price remains important for the further expansion of the functional food market [137].

Advertisements for supplemental product marketing are the most dominant source of information on functional food. Trust in the mass media and peer recommendations remarkably govern purchase intentions for these products [138]. It is important to note that the effects of “superfoods” (a term commonly used in marketing) require scientific proof [139]. Governments should incentivize companies dedicated to functional food research, and health claims should be supported by in vitro, animal, and clinical trials. Collaboration between food scientists and professionals from other fields is important in order to gain a comprehensive understanding of the impact of functional foods on human metabolism [137]. Public health education activities organized through digital health communication media are also important for increasing the understanding of the consumer community [140].

The current study also identified many modifiable risk factors that can greatly influence the development of GERD disease [11]. Obesity is a known risk factor for GERD, as excess weight can exert pressure on the stomach and promote acid reflux [141,142]. Functional foods that support healthy weight management can be beneficial in managing symptoms [143,144].

More randomized controlled trials and clinical and epidemiological studies are needed to investigate the possible mechanisms behind functional foods’ effects on GERD in terms of prevention and treatment [145]. Public policy and educational approaches regarding lifestyle changes in relation to functional food consumption are required to further enhance the benefits for GERD treatment. Well-designed randomized controlled trials are needed further to study the effects of dietary therapy on GERD management as non-pharmacological options continue to gain popularity.

## 6. Conclusions

In conclusion, consumers in the 21st century must face increasing risks related to environmental pollution, stress, social challenges, and health problems. Functional products have the potential to help improve physical and mental health, leading to higher quality of life. Functional foods can help improve GERD symptoms by reducing acid reflux episodes, protecting the esophagus, enhancing digestive function, and alleviating inflammation. Personalizing diets based on symptoms and focusing on factors like meal size, timing, and macronutrient composition are more effective than elimination diets. Exploring non-pharmacological options can lead to better treatment strategies for GERD. Further research is needed through well-designed trials to better understand dietary therapy’s effects on GERD management.

## Data Availability

Not applicable.

## References

[1] Ozkahraman Kırık M., Uslu Coskun B., Cingi C., Yorgancıoğlu A., Bayar Muluk N., Cruz A.A. (2023). Gastroesophageal Reflux Disease BT—Airway Diseases.

[2] Durazzo M., Lupi G., Cicerchia F., Ferro A., Barutta F., Beccuti G., Gruden G., Pellicano R. (2020). Extra-Esophageal Presentation of Gastroesophageal Reflux Disease: 2020 Update. J. Clin. Med..

[3] Chhabra P., Ingole N. (2022). Gastroesophageal Reflux Disease (GERD): Highlighting Diagnosis, Treatment, and Lifestyle Changes. Cureus.

[4] Širić L., Rosso M., Včev A., Neri V., Ahmed M. (2021). Extraesophageal Manifestations and Symptoms of Esophageal Diseases. Esophagitis and Gastritis.

[5] Baker M.T., Lu P., Parrella J.A., Leggette H.R. (2022). Consumer Acceptance toward Functional Foods: A Scoping Review. Int. J. Environ. Res. Public Health.

[6] Newberry C., Lynch K. (2019). The role of diet in the development and management of gastroesophageal reflux disease: Why we feel the burn. J. Thorac. Dis..

[7] Yadlapati R., Hubscher E., Pelletier C., Jacob R., Brackley A., Shah S. (2022). Induction and maintenance of healing in erosive esophagitis in the United States. Expert Rev. Gastroenterol. Hepatol..

[8] Meyiz H., El Agheb M., Lamine A., El Yousfi M., Aqodad N., Benajeh D., El Abkari M., Ibrahimi A., Mellouki I. (2019). The Impact of Gastroesophageal Reflux on the Quality of Life: About a Series of 100 Patients at Fez University Hospital. Open J. Gastroenterol..

[9] Gorczyca R., Pardak P., Pękala A., Filip R. (2019). Impact of gastroesophageal reflux disease on the quality of life of Polish patients. World J. Clin. Cases.

[10] Alshammari S.A., Alabdulkareem A.M., Aloqeely K.M., Alhumud M.I., Alghufaily S.A., Al-Dossare Y.I., Alrashdan N.O. (2020). The Determinants of the Quality of Life of Gastroesophageal Reflux Disease Patients Attending King Saud University Medical City. Cureus.

[11] Rasool M.F., Sarwar R., Arshad M.S., Imran I., Saeed H., Majeed A., Akbar M., Chaudhry M.O., Rehman A.U., Ashraf W. (2021). Assessing the frequency and risk factors associated with gastroesophageal reflux disease (GERD) in southern Punjab, Pakistan. Risk Manag. Healthc. Policy.

[12] Yuan S., Larsson S.C. (2022). Adiposity, diabetes, lifestyle factors and risk of gastroesophageal reflux disease: A Mendelian randomization study. Eur. J. Epidemiol..

[13] Kariri A.M., Darraj M.A., Wassly A., Arishi H.A., Lughbi M., Kariri A., Madkhali A.M., Ezzi M.I., Khawaji B. (2020). Prevalence and Risk Factors of Gastroesophageal Reflux Disease in Southwestern Saudi Arabia. Cureus.

[14] Islam S.M.R., Siddiqua T.J. (2020). 20—Functional Foods in Cancer Prevention and Therapy: Recent Epidemiological Findings. Functional Foods in Cancer Prevention and Therapy.

[15] Fan W.J., Hou Y.T., Sun X.H., Li X.Q., Wang Z.F., Guo M., Zhu L.M., Wang N., Yu K., Li J.N. (2018). Effect of high-fat, standard, and functional food meals on esophageal and gastric pH in patients with gastroesophageal reflux disease and healthy subjects. J. Dig. Dis..

[16] Antunes C., Aleem A., Curtis S.A. (2023). Gastroesophageal Reflux Disease. StatPearls [Internet].

[17] Rettura F., Bronzini F., Campigotto M., Lambiase C., Pancetti A., Berti G., Marchi S., de Bortoli N., Zerbib F., Savarino E. (2021). Refractory Gastroesophageal Reflux Disease: A Management Update. Front. Med..

[18] Zheng Z., Shang Y., Wang N., Liu X., Xin C., Yan X., Zhai Y., Yin J., Zhang J., Zhang Z. (2021). Current Advancement on the Dynamic Mechanism of Gastroesophageal Reflux Disease. Int. J. Biol. Sci..

[19] Carabotti M., Severi C. (2017). Chapter 38—Upper Gastrointestinal Diseases before and after Bariatric Surgery. Metabolism and Pathophysiology of Bariatric Surgery.

[20] Rodriguez L., Nurko S. (2021). 64—Gastrointestinal Motility Procedures. Pediatric Gastrointestinal and Liver Disease.

[21] Chen R.R., Chen Q.Z., Feng B.C., Wang M.F., Lin L., Ye B.X., Jiang L.Q. (2023). Characteristics of reflux and gastric electrical activity in gastroesophageal reflux disease with ineffective esophageal motility. J. Dig. Dis..

[22] MacFarlane B. (2018). Management of gastroesophageal reflux disease in adults: A pharmacist’s perspective. Integr. Pharm. Res. Pract..

[23] Wu J., Liu D., Feng C., Luo Y., Nian Y., Wang X., Zhang J. (2018). The Characteristics of Postprandial Proximal Gastric Acid Pocket in Gastroesophageal Reflux Disease. Med. Sci. Monit..

[24] Sumi S., Ishimura N., Mikami H., Okimoto E., Tamagawa Y., Mishiro T., Kinoshita Y., Ishihara S. (2021). Evaluations of Gastric Acid Pocket Using Novel Vertical 8-Channel pH Monitoring System and Effects of Acid Secretion Inhibitors. J. Neurogastroenterol. Motil..

[25] Eriksson S.E., Zheng P., Sarici I.S., Shen X., Jobe B.A., Ayazi S. (2023). The impact of delayed gastric emptying as measured by gastric emptying scintigraphy on the outcome of magnetic sphincter augmentation. Surg. Endosc..

[26] Dunn C.P., Wu J., Gallagher S.P., Putnam L.R., Bildzukewicz N.A., Lipham J.C. (2021). Understanding the GERD Barrier. J. Clin. Gastroenterol..

[27] Schwinghammer T.L., DiPiro J.T., Ellingrod V.L., DiPiro C.V. (2021). Gastroesophageal Reflux Disease. Pharmacotherapy Handbook.

[28] Patel D.A., Yadlapati R., Vaezi M.F. (2022). Esophageal Motility Disorders: Current Approach to Diagnostics and Therapeutics. Gastroenterology.

[29] Abdelghani A., Ibrahim A., El-Sayed E.S., El Sherbiny M., Al-Badry A. (2023). Esophageal motility disorders in symptomatic patients and its relation to age. BMC Gastroenterol..

[30] Agostinis C., Bossi F., Mangogna A., Balduit A., Pacor M., Giacomello E., Belmonte B., Greco D., Rodolico V., Voinovich D. (2020). Protective and regenerative effects of a novel medical device against esophageal mucosal damage using in vitro and ex vivo models. Biomed. Pharmacother..

[31] Ustaoglu A., Nguyen A., Spechler S., Sifrim D., Souza R., Woodland P. (2020). Mucosal pathogenesis in gastro-esophageal reflux disease. Neurogastroenterol. Motil..

[32] Sfara A., Dumitrascu D.L. (2019). The management of hiatal hernia: An update on diagnosis and treatment. Med. Pharm. Rep..

[33] Zheng Z., Liu X., Xin C., Zhang W., Gao Y., Zeng N., Li M., Cai J., Meng F., Liu D. (2021). A new technique for treating hiatal hernia with gastroesophageal reflux disease: The laparoscopic total left-side surgical approach. BMC Surg..

[34] Sharma P., Yadlapati R. (2021). Pathophysiology and treatment options for gastroesophageal reflux disease: Looking beyond acid. Ann. N. Y. Acad. Sci..

[35] Marabotto E., Savarino V., Ghisa M., Frazzoni M., Ribolsi M., Barberio B., Savarino E. (2022). Advancements in the use of 24-hour impedance-pH monitoring for GERD diagnosis. Curr. Opin. Pharmacol..

[36] Czarniak P., Ahmadizar F., Hughes J., Parsons R., Kavousi M., Ikram M., Stricker B.H. (2022). Proton pump inhibitors are associated with incident type 2 diabetes mellitus in a prospective population-based cohort study. Br. J. Clin. Pharmacol..

[37] Robinson B.J. (2020). Regular use of PPIs linked with increased risk of type 2 diabetes, study suggests. Pharm. J..

[38] Rajput M.A., Ali F., Zehra T., Zafar S., Kumar G. (2020). The effect of proton pump inhibitors on glycaemic control in diabetic patients. J. Taibah Univ. Med. Sci..

[39] Sun X.-M., Tan J.-C., Zhu Y., Lin L. (2015). Association between diabetes mellitus and gastroesophageal reflux disease: A meta-analysis. World J. Gastroenterol..

[40] Althoff M.D., Sharma S. (2023). Gastroesophageal Reflux, Atopic Dermatitis, and Asthma: Finally Evidence for Causal Links?. Am. J. Respir. Crit. Care Med..

[41] Grandes X.A., Talanki Manjunatha R., Habib S., Sangaraju S.L., Yepez D. (2022). Gastroesophageal Reflux Disease and Asthma: A Narrative Review. Cureus.

[42] Lin H.-C., Xirasagar S., Chung S.-D., Huang C.-C., Tsai M.-C., Chen C.-H. (2017). Fewer acute respiratory infection episodes among patients receiving treatment for gastroesophageal reflux disease. PLoS ONE.

[43] Sharma P. (2022). Barrett Esophagus: A Review. JAMA.

[44] Souza R.F., Spechler S.J. (2022). Mechanisms and pathophysiology of Barrett oesophagus. Nat. Rev. Gastroenterol. Hepatol..

[45] Mahajan R., Kulkarni R., Stoopler E.T. (2022). Gastroesophageal reflux disease and oral health: A narrative review. Spec. Care Dent. Off. Publ. Am. Assoc. Hosp. Dent. Acad. Dent. Handicap. Am. Soc. Geriatr. Dent..

[46] Shah S. (2020). COVID-19 and paediatric dentistry- traversing the challenges. A narrative review. Ann. Med. Surg..

[47] Chakraborty A., Anjankar A.P. (2022). Association of Gastroesophageal Reflux Disease with Dental Erosion. Cureus.

[48] Howard J.P., Howard L.J., Geraghty J., Leven A.J., Ashley M. (2023). Gastrointestinal conditions related to tooth wear. Br. Dent. J..

[49] Grossi L., Ciccaglione A.F., Marzio L. (2017). World Journal of Gastroenterology. World J. Gastroenterol..

[50] Heidarzadeh-Esfahani N., Soleimani D., Hajiahmadi S., Moradi S., Heidarzadeh N., Nachvak S.M. (2021). Dietary Intake in Relation to the Risk of Reflux Disease: A Systematic Review. Prev. Nutr. Food Sci..

[51] Chamorro R., Kannenberg S., Wilms B., Kleinerüschkamp C., Meyhöfer S., Park S.Q., Lehnert H., Oster H., Meyhöfer S.M. (2022). Meal Timing and Macronutrient Composition Modulate Human Metabolism and Reward-Related Drive to Eat. Nutrients.

[52] Fox M., Gyawali C.P. (2023). Dietary factors involved in GERD management. Best Pract. Res. Clin. Gastroenterol..

[53] Wildi S.M., Tutuian R., Castell D.O. (2004). The Influence of Rapid Food Intake on Postprandial Reflux: Studies in Healthy Volunteers. Off. J. Am. Coll. Gastroenterol. | ACG.

[54] Mangano M., Colombo P., Bianchi P.A., Penagini R. (2002). Fat and Esophageal Sensitivity to Acid. Dig. Dis. Sci..

[55] Li Y., Su Z., Li P., Li Y., Johnson N., Zhang Q., Du S., Zhao H., Li K., Zhang C. (2020). Association of Symptoms with Eating Habits and Food Preferences in Chronic Gastritis Patients: A Cross-Sectional Study. Evid.-Based Complement. Altern. Med..

[56] Fox M., Barr C., Nolan S., Lomer M., Anggiansah A., Wong T. (2007). The effects of dietary fat and calorie density on esophageal acid exposure and reflux symptoms. Clin. Gastroenterol. Hepatol. Off. Clin. Pract. J. Am. Gastroenterol. Assoc..

[57] Zhang M., Hou Z.-K., Huang Z.-B., Chen X.-L., Liu F.-B. (2021). Dietary and Lifestyle Factors Related to Gastroesophageal Reflux Disease: A Systematic Review. Ther. Clin. Risk Manag..

[58] Beigrezaei S., Sasanfar B., Nafei Z., Behniafard N., Aflatoonian M., Salehi-Abargouei A. (2023). Dietary approaches to stop hypertension (DASH)-style diet in association with gastroesophageal reflux disease in adolescents. BMC Public Health.

[59] Keshteli A.H., Shaabani P., Tabibian S.-R., Saneei P., Esmaillzadeh A., Adibi P. (2017). The relationship between fruit and vegetable intake with gastroesophageal reflux disease in Iranian adults. J. Res. Med. Sci. Off. J. Isfahan Univ. Med. Sci..

[60] Yuan L.Z., Yi P., Wang G.S., Tan S.Y., Huang G.M., Qi L.Z., Jia Y., Wang F. (2019). Lifestyle intervention for gastroesophageal reflux disease: A national multicenter survey of lifestyle factor effects on gastroesophageal reflux disease in China. Therap. Adv. Gastroenterol..

[61] Myung S.-K., Kim H.-B., Lee Y.-J., Choi Y.-J., Oh S.-W. (2021). Calcium Supplements and Risk of Cardiovascular Disease: A Meta-Analysis of Clinical Trials. Nutrients.

[62] Morelli M.B., Santulli G., Gambardella J. (2020). Calcium supplements: Good for the bone, bad for the heart? A systematic updated appraisal. Atherosclerosis.

[63] Özenoğlu A., Anul N., Özçelikçi B. (2023). The relationship of gastroesophageal reflux with nutritional habits and mental disorders. Hum. Nutr. Metab..

[64] Basharat S., Binté F., Amir S., Zubair M. (2020). Association of Dietary Practices and Lifestyle Modifications in Gastroesophageal Reflux Disease in Pakistani Women. Am. Sci. Res. J. Eng. Technol. Sci..

[65] Chen J.W., Vela M.F., Peterson K.A., Carlson D.A. (2023). AGA Clinical Practice Update on the Diagnosis and Management of Extraesophageal Gastroesophageal Reflux Disease: Expert Review. Clin. Gastroenterol. Hepatol..

[66] Lee D.P., Chang K.J. (2022). Endoscopic Management of GERD. Dig. Dis. Sci..

[67] Ahmed M.H., Vasas D., Hassan A., Molnár J. (2022). The impact of functional food in prevention of malnutrition. PharmaNutrition.

[68] Guadagnoli L., Simons M., McGarva J., Taft T.H., van Tilburg M.A.L. (2022). Improving Patient Adherence to Lifestyle Changes for the Management of Gastroesophageal Reflux. Patient Prefer. Adherence.

[69] Wilks A., Panahi L., Udeani G., Surani S., Chai J. (2022). Review of Gastroesophageal Reflux Pharmacotherapy Management. Gastroesophageal Reflux Disease.

[70] Kröner P.T., Cortés P., Lukens F.J. (2021). The Medical Management of Gastroesophageal Reflux Disease: A Narrative Review. J. Prim. Care Community Health.

[71] Turaga A.H., Salem Y.H. (2023). Transoral incisionless fundoplication and open hiatal hernia repair: A case report. Front. Gastroenterol..

[72] Bell R.C.W. (2021). Beyond Proton Pump Inhibitors and Nissen Fundoplication: Minimally Invasive Alternatives for Gastroesophageal Reflux Disease. Gastroenterol. Hepatol..

[73] Dumitru V., Hoara P., Dumitru D., Birla R., Gindea C., Constantinoiu S. (2020). Invasive Treatment Options for Gastro-Esophageal Reflux Disease. J. Med. Life.

[74] Hoffsten J., Forsell Y. (2022). Laparoscopic Nissen fundoplication versus 90° and 180° fundoplication for gastroesophageal reflux disease: Systematic review and meta-analysis of 5-year outcomes. Eur. Surg.—Acta Chir. Austriaca.

[75] Loo G.H., Rajan R., Deva Tata M., Ritza Kosai N. (2020). Changes in the disease-specific quality of life following Dor fundoplication. A multicentre cross-sectional study. Ann. Med. Surg..

[76] Tjeerdsma M., Quinn K.R., Helmer S.D., Vincent K.B. (2022). Comparing Outcomes of Robotic-Assisted versus Conventional Laparoscopic Hiatal Hernia Repair. Kansas J. Med..

[77] Ali A., Rahut D.B. (2019). Healthy Foods as Proxy for Functional Foods: Consumers’ Awareness, Perception, and Demand for Natural Functional Foods in Pakistan. Int. J. Food Sci..

[78] Vettorazzi A., López de Cerain A., Sanz-Serrano J., Gil A.G., Azqueta A. (2020). European Regulatory Framework and Safety Assessment of Food-Related Bioactive Compounds. Nutrients.

[79] Arnold M., Rajagukguk Y.V., Gramza-Michałowska A. (2021). Functional food for elderly high in antioxidant and chicken eggshell calcium to reduce the risk of osteoporosis—A narrative review. Foods.

[80] González-Díaz C., Vilaplana-Aparicio M.J., Iglesias-García M. (2020). How Is Functional Food Advertising Understood? An Approximation in University Students. Nutrients.

[81] Siró I., Kápolna E., Kápolna B., Lugasi A. (2008). Functional food. Product development, marketing and consumer acceptance—A review. Appetite.

[82] Budreviciute A., Damiati S., Sabir D.K., Onder K., Schuller-Goetzburg P., Plakys G., Katileviciute A., Khoja S., Kodzius R. (2020). Management and Prevention Strategies for Non-communicable Diseases (NCDs) and Their Risk Factors. Front. Public Health.

[83] Zhang T., Zhang B., Tian W., Wei Y., Wang F., Yin X., Wei X., Liu J., Tang X. (2022). Trends in gastroesophageal reflux disease research: A bibliometric and visualized study. Front. Med..

[84] Aman F., Masood S. (2020). How Nutrition can help to fight against COVID-19 Pandemic. Pakistan J. Med. Sci..

[85] Topolska K., Florkiewicz A., Filipiak-Florkiewicz A. (2021). Functional Food-Consumer Motivations and Expectations. Int. J. Environ. Res. Public Health.

[86] Gu S., Ślusarczyk B., Hajizada S., Kovalyova I., Sakhbieva A. (2021). Impact of the COVID-19 pandemic on online consumer purchasing behavior. J. Theor. Appl. Electron. Commer. Res..

[87] Schifferstein H.N.J., de Boer A., Lemke M. (2021). Conveying information through food packaging: A literature review comparing legislation with consumer perception. J. Funct. Foods.

[88] Wang H., Ab Gani M.A.A., Liu C. (2023). Impact of Snack Food Packaging Design Characteristics on Consumer Purchase Decisions. SAGE Open.

[89] Nguyen N., Nguyen H.V., Nguyen P.T., Tran V.T., Nguyen H.N., Nguyen T.M.N., Cao T.K., Nguyen T.H. (2019). Some Key Factors Affecting Consumers’ Intentions to Purchase Functional Foods: A Case Study of Functional Yogurts in Vietnam. Foods.

[90] FDA (2004). Label Claim for Food & Dietary Supplements.

[91] Fernández-Ríos A., Laso J., Hoehn D., Amo-Setién F.J., Abajas-Bustillo R., Ortego C., Fullana-i-Palmer P., Bala A., Batlle-Bayer L., Balcells M. (2022). A critical review of superfoods from a holistic nutritional and environmental approach. J. Clean. Prod..

[92] Jagdale Y.D., Mahale S.V., Zohra B., Nayik G.A., Dar A.H., Ali Khan K., Abdi G., Karabagias I.K. (2021). Nutritional profile and potential health benefits of super foods: A review. Sustainability.

[93] Santillán-Urquiza E., Ruiz-Espinosa H., Angulo-Molina A., Vélez Ruiz J.F., Méndez-Rojas M.A. (2017). 8—Applications of nanomaterials in functional fortified dairy products: Benefits and implications for human health. Nutrient Delivery.

[94] Sadler C.R., Grassby T., Hart K., Raats M., Sokolović M., Timotijevic L. (2021). Processed food classification: Conceptualisation and challenges. Trends Food Sci. Technol..

[95] Smyth S.J. (2020). The human health benefits from GM crops. Plant Biotechnol. J..

[96] Martirosyan D., Lampert T., Lee M. (2022). A comprehensive review on the role of food bioactive compounds in functional food science. Funct. Food Sci..

[97] Muncke J., Andersson A.M., Backhaus T., Boucher J.M., Carney Almroth B., Castillo Castillo A., Chevrier J., Demeneix B.A., Emmanuel J.A., Fini J.B. (2020). Impacts of food contact chemicals on human health: A consensus statement. Environ. Health A Glob. Access Sci. Source.

[98] Yuan S., Li C., Zhang Y., Yu H., Xie Y., Guo Y., Yao W. (2021). Ultrasound as an emerging technology for the elimination of chemical contaminants in food: A review. Trends Food Sci. Technol..

[99] Liu R.H. (2013). Health-promoting components of fruits and vegetables in the diet. Adv. Nutr..

[100] Martínez E., Pardo J.E., Rabadán A., Álvarez-Ortí M. (2023). Effects of Animal Fat Replacement by Emulsified Melon and Pumpkin Seed Oils in Deer Burgers. Foods.

[101] Astrup A., Magkos F., Bier D.M., Brenna J.T., de Oliveira Otto M.C., Hill J.O., King J.C., Mente A., Ordovas J.M., Volek J.S. (2020). Saturated Fats and Health: A Reassessment and Proposal for Food-Based Recommendations: JACC State-of-the-Art Review. J. Am. Coll. Cardiol..

[102] Wang Z., Mhaske P., Farahnaky A., Kasapis S., Majzoobi M. (2022). Cassava starch: Chemical modification and its impact on functional properties and digestibility, a review. Food Hydrocoll..

[103] Arshad R., Gulshad L., Haq I.U., Farooq M.A., Al-Farga A., Siddique R., Manzoor M.F., Karrar E. (2021). Nanotechnology: A novel tool to enhance the bioavailability of micronutrients. Food Sci. Nutr..

[104] Xiong K., Zhou L., Wang J., Ma A., Fang D., Xiong L., Sun Q. (2020). Construction of food-grade pH-sensitive nanoparticles for delivering functional food ingredients. Trends Food Sci. Technol..

[105] Putnik P., Kovačević D.B. (2021). Sustainable functional food processing. Foods.

[106] Knorr D., Augustin M.A., Tiwari B. (2020). Advancing the Role of Food Processing for Improved Integration in Sustainable Food Chains. Front. Nutr..

[107] Kasote D., Tiozon R.N., Sartagoda K.J.D., Itagi H., Roy P., Kohli A., Regina A., Sreenivasulu N. (2021). Food Processing Technologies to Develop Functional Foods with Enriched Bioactive Phenolic Compounds in Cereals. Front. Plant Sci..

[108] Morozov S., Isakov V., Konovalova M. (2018). Fiber-enriched diet helps to control symptoms and improves esophageal motility in patients with non-erosive gastroesophageal reflux disease. World J. Gastroenterol..

[109] Hong S.W., Chun J., Park S., Lee H.J., Im J.P., Kim J.S. (2018). Aloe vera Is Effective and Safe in Short-term Treatment of Irritable Bowel Syndrome: A Systematic Review and Meta-analysis. J. Neurogastroenterol. Motil..

[110] Kontareva V.Y., Belik S.N., Morgul E.V., Gorlov I.F., Slozhenkina M.I. (2020). Yogurt enriched to correct intestinal microflora in dysbiosis. IOP Conf. Ser. Earth Environ. Sci..

[111] Zhai Z., Wang J., Huang B., Yin S. (2019). Low-fat yogurt alleviates the pro-inflammatory cytokine IL-1β-induced intestinal epithelial barrier dysfunction. J. Dairy Sci..

[112] Ioniță-Mîndrican C.-B., Ziani K., Mititelu M., Oprea E., Neacșu S.M., Moroșan E., Dumitrescu D.-E., Roșca A.C., Drăgănescu D., Negrei C. (2022). Therapeutic Benefits and Dietary Restrictions of Fiber Intake: A State of the Art Review. Nutrients.

[113] Muliawati E., Carolin B.T., Lail N.H. (2022). Comparison Between the Provision of White Ambon Banana Fruit and Red Dragon Fruit on Hemoglobin Levels. Nurs. Health Sci. J..

[114] Zou F., Tan C., Zhang B., Wu W., Shang N. (2022). The Valorization of Banana By-Products: Nutritional Composition, Bioactivities, Applications, and Future Development. Foods.

[115] Nikkhah Bodagh M., Maleki I., Hekmatdoost A. (2019). Ginger in gastrointestinal disorders: A systematic review of clinical trials. Food Sci. Nutr..

[116] Schulz R.M., Ahuja N.K., Slavin J.L. (2022). Effectiveness of Nutritional Ingredients on Upper Gastrointestinal Conditions and Symptoms: A Narrative Review. Nutrients.

[117] Valussi M. (2012). Functional foods with digestion-enhancing properties. Int. J. Food Sci. Nutr..

[118] Fiorini G., Saracino I.M., Pavoni M., Saccomanno L., Vaira D. (2020). Efficacy of a new nutraceutical formulation (CHETOGERD(^®^)) in patients with nonerosive reflux disease (NERD): A prospective observational study. Intern. Emerg. Med..

[119] Valdes A.M., Walter J., Segal E., Spector T.D. (2018). Role of the gut microbiota in nutrition and health. BMJ.

[120] Wang X., Zhang P., Zhang X. (2021). Probiotics Regulate Gut Microbiota: An Effective Method to Improve Immunity. Molecules.

[121] Dahiya D., Nigam P.S. (2022). The Gut Microbiota Influenced by the Intake of Probiotics and Functional Foods with Prebiotics Can Sustain Wellness and Alleviate Certain Ailments like Gut-Inflammation and Colon-Cancer. Microorganisms.

[122] Das T.K., Pradhan S., Chakrabarti S., Mondal K.C., Ghosh K. (2022). Current status of probiotic and related health bene-fits. Appl. Food Res..

[123] Cheng J., Ouwehand A.C. (2020). Gastroesophageal Reflux Disease and Probiotics: A Systematic Review. Nutrients.

[124] Rollet M., Bohn T., Vahid F., ORISCAV Working Group (2021). Association between Dietary Factors and Constipation in Adults Living in Luxembourg and Taking Part in the ORISCAV-LUX 2 Survey. Nutrients.

[125] Heidelbaugh J.J., Nostrant T.T., Kim C., Van Harrison R. (2003). Management of gastroesophageal reflux disease. Am. Fam. Physician.

[126] Hamid N.K.A., Somdare P.O., Md Harashid K.A., Othman N.A., Kari Z.A., Wei L.S., Dawood M.A.O. (2022). Effect of papaya (Carica papaya) leaf extract as dietary growth promoter supplement in red hybrid tilapia (Oreochromis mossambicus × Oreochromis niloticus) diet. Saudi J. Biol. Sci..

[127] Kostiuchenko O., Kravchenko N., Markus J., Burleigh S., Fedkiv O., Cao L., Letasiova S., Skibo G., Fåk Hållenius F., Prykhodko O. (2022). Effects of Proteases from Pineapple and Papaya on Protein Digestive Capacity and Gut Microbiota in Healthy C57BL/6 Mice and Dose-Manner Response on Mucosal Permeability in Human Reconstructed Intestinal 3D Tissue Model. Metabolites.

[128] Peng Y., Ao M., Dong B., Jiang Y., Yu L., Chen Z., Hu C., Xu R. (2021). Anti-Inflammatory Effects of Curcumin in the Inflammatory Diseases: Status, Limitations and Countermeasures. Drug Des. Devel. Ther..

[129] Sharifi-Rad J., Rayess Y.E., Rizk A.A., Sadaka C., Zgheib R., Zam W., Sestito S., Rapposelli S., Neffe-Skocińska K., Zielińska D. (2020). Turmeric and Its Major Compound Curcumin on Health: Bioactive Effects and Safety Profiles for Food, Pharmaceutical, Biotechnological and Medicinal Applications. Front. Pharmacol..

[130] Kwiecien S., Magierowski M., Majka J., Ptak-Belowska A., Wójcik-Grzybek D., Sliwowski Z., Magierowska K., Brzozowski T. (2019). Curcumin: A Potent Protectant against Esophageal and Gastric Disorders. Int. J. Mol. Sci..

[131] Santos H.O., May T.L., Bueno A.A. (2023). Eating more sardines instead of fish oil supplementation: Beyond omega-3 polyunsaturated fatty acids, a matrix of nutrients with cardiovascular benefits. Front. Nutr..

[132] Oh D.Y., Walenta E. (2014). Omega-3 Fatty Acids and FFAR4. Front. Endocrinol..

[133] Fabisiak A., Bartoszek A., Talar M., Binienda A., Dziedziczak K., Krajewska J.B., Mosińska P., Niewinna K., Tarasiuk A., Mokrowiecka A. (2020). Expression of FFAR3 and FFAR4 Is Increased in Gastroesophageal Reflux Disease. J. Clin. Med..

[134] Ozkur M., Benlier N., Takan I., Vasileiou C., Georgakilas A.G., Pavlopoulou A., Cetin Z., Saygili E.I. (2022). Ginger for Healthy Ageing: A Systematic Review on Current Evidence of Its Antioxidant, Anti-Inflammatory, and Anticancer Properties. Oxid. Med. Cell. Longev..

[135] Sharma S., Shukla M.K., Sharma K.C., Tirath, Kumar L., Anal J.M.H., Upadhyay S.K., Bhattacharyya S., Kumar D. (2023). Revisiting the therapeutic potential of gingerols against different pharmacological activities. Naunyn. Schmiedebergs. Arch. Pharmacol..

[136] Kim K.J., Kim S.H., Shin M.-R., Kim Y.J., Park H.-J., Roh S.-S. (2019). Protective effect of S-allyl cysteine-enriched black garlic on reflux esophagitis in rats via NF-κB signaling pathway. J. Funct. Foods.

[137] Granato D., Barba F.J., Bursać Kovačević D., Lorenzo J.M., Cruz A.G., Putnik P. (2020). Functional Foods: Product Development, Technological Trends, Efficacy Testing, and Safety. Annu. Rev. Food Sci. Technol..

[138] Huang L., Bai L., Gong S. (2020). The effects of carrier, benefit, and perceived trust in information channel on functional food purchase intention among Chinese consumers. Food Qual. Prefer..

[139] Konstantinidi M., Koutelidakis A.E. (2019). Functional Foods and Bioactive Compounds: A Review of Its Possible Role on Weight Management and Obesity’s Metabolic Consequences. Medicines.

[140] Sumaedi S., Sumardjo, Saleh A., Syukri A.F. (2022). Factors influencing males’ loyalty toward functional foods during the COVID-19 pandemic. Int. J. Public Health Sci..

[141] Thalheimer A., Bueter M. (2021). Excess Body Weight and Gastroesophageal Reflux Disease. Visc. Med..

[142] Takahashi K., Seki Y., Kasama K., Amiki M., Baba S., Ito M., Tanaka T., Kanehira E. (2020). Prevalence of reflux esophagitis in obese Japanese undergoing bariatric surgery. JGH Open.

[143] Green M., Arora K., Prakash S. (2020). Microbial Medicine: Prebiotic and Probiotic Functional Foods to Target Obesity and Metabolic Syndrome. Int. J. Mol. Sci..

[144] Sandner G., König A., Wallner M., Weghuber J. (2020). Functional foods—Dietary or herbal products on obesity: Application of selected bioactive compounds to target lipid metabolism. Curr. Opin. Food Sci..

[145] Gupta E., Mishra P. (2021). Functional Food with Some Health Benefits, So Called Superfood: A Review. Curr. Nutr. Food Sci..

